# The global epidemiology of clonorchiasis and its relation with cholangiocarcinoma

**DOI:** 10.1186/2049-9957-1-4

**Published:** 2012-10-25

**Authors:** Men-Bao Qian, Ying-Dan Chen, Song Liang, Guo-Jing Yang, Xiao-Nong Zhou

**Affiliations:** 1 National Institute of Parasitic Diseases, Chinese Center for Disease Control and Prevention; WHO Collaborative Center for Malaria, Schistosomiasis and Filariasis; Key Laboratory of Parasite and Vector Biology, Ministry of Health, Shanghai, People’s Republic of China; 2 Department of Environmental and Global Health, College of Public Health and Health Professions, and Emerging Pathogens Institute, University of Florida, Gainesville, USA; 3 Jiangsu Institute of Parasitic Diseases, Wuxi, People’s Republic of China; 4 School of Public Health, Chinese University of Hong Kong, Hong Kong, People’s Republic of China

**Keywords:** Clonorchiasis, *Clonorchis sinensis*, Epidemiology, Cholangiocarcinoma, Odds ratio

## Abstract

This paper reviews the epidemiological status and characteristics of clonorchiasis at global level and the etiological relationship between *Clonorchis sinensis* infection and cholangiocarcinoma (CCA). A conservative estimation was made that 15 million people were infected in the world in 2004, of which over 85% distributed in China. The epidemiology of clonorchiasis is characterized by rising trend in its prevalence, variability among sexes and age, as well as endemicity in different regions. More data indicate that *C. sinensis* infection is carcinogenic to human, and it is predicted that nearly 5 000 CCA cases attributed to *C. sinensis* infection may occur annually in the world decades later, with its overall odds ratio of 4.47. Clonorchiasis is becoming one major public health problem in east Asia, and it is worthwhile to carry out further epidemiological studies.

## Multilingual abstracts

Please see Additional file 1 for translations of the abstract into the six official working languages of the United Nations.

## Review

**Figure 1:**
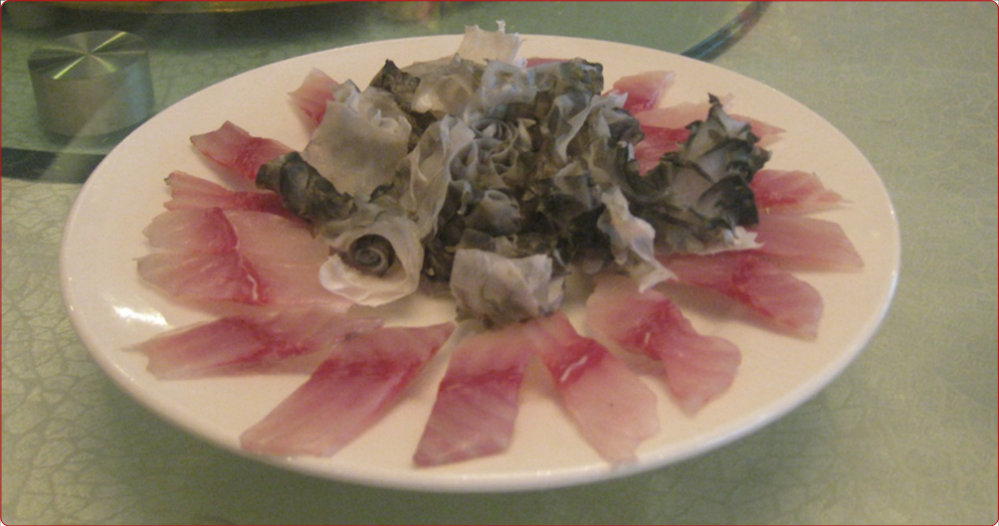
**Raw freshwater fish consumed by human beings.** The outer layer is raw flesh which has not been cooked anyway, while the inner one is skin of fish which has only been blanched in hot water within a few seconds.

Liver flukes are a polyphyletic group of trematodes (phylum Platyhelminthes), including *Clonorchis sinensis*, *Opisthorchis viverrini* and *Opisthorchis felineus* from family opisthorchiidae and *Fasciola* spp. from family fasciolidae [1-4]. Here, opisthorchiidae, especially *C. sinensis* is focused on, so liver flukes are specially termed to this family. Adults of liver flukes, localizing in the liver of various mammals including humans, produce eggs which are passed into the intestine. Most of the parasites live in bile ducts, gallbladder and liver parenchyma, causing liver and biliary diseases. Human beings are infected through ingestion of raw or undercooked fish which contains the metacercariae of liver flukes (Figure 1) [1-4].

Cholangiocarcinoma (CCA) is a cancer of the bile ducts. Recent evidences support the fact that CCA is the most severe complication of liver fluke infection, and *C. sinensis* and *O. viverrini* infections are both classified as “carcinogenic to humans” (Group 1) by the International Agency for Research on Cancer (IARC) in 2009 [5,6].

Due to the absence of definite information on geographical distribution and disease burden, their public health impact has been underestimated for a long time [7]. Here we review the global epidemiological status and characteristics of clonorchiasis and the etiological relationship between *C. sinensis* infection and CCA.

## Epidemiology of clonorchiasis

### Distribution

Clonorchiasis is predominantly endemic in east Asia, but it may also occur in other regions where there are immigrants from endemic areas [8]. Due to the growth and movement of population and the rapid development of aquaculture, the fact of clonorchiasis hindering the local economic development is increasingly notified [2,7].

Three large-scale surveys for clonorchiasis have been carried out in mainland of China. The prevalence of clonorchiasis was 0.37% according to the first national parasite survey involving in 30 Provinces/Municipalities/Autonomous Regions (P/M/As) between 1988 and 1992 (hereinafter referring as 1992) [9]. Then, it increased to 0.58% in the second national parasite survey involving in 31 P/M/As between 2001 and 2004 (hereinafter referring as 2004a) [10,11]. Meanwhile, another special survey for clonorchiasis in 27 endemic P/M/As was carried out between 2002 and 2004 (hereinafter referring as 2004b), which showed the prevalence was 2.40% and the population infected were 12.49 million [10,11]. The 2004b special survey aimed at clonorchiasis and three Kato-Katz smears were examined for each fecal sample, while the national surveys didn’t focus on *C. sinensis* but rather detected any parasite species in a single Kato-Katz smear. Hence, result from the special survey was more reasonable. Clonorchiasis was also endemic in Taiwan, mainly located in Miao-li in the north, Sun-moon Lake in the middle and Mei-nung in the south [12,13] and a prevalence of 0.4%-1.0% was estimated [1]. Thus, a conservative estimation was made that 89 000 people were infected in 2004 among the total population of 22.28 million [14]. Based on the hospitalized cases in two hospitals in Hong Kong between 1990 and 1997, out of the 1 782 cases with intestinal parasitic diseases, 1 162 (65.21%) were infected with *C. sinensis*[15]. According to the estimation by WHO in 1995, the population infected in Hong Kong was 333 000 [8]. From 1958 to 1991, five local surveys were implemented in Macao and the prevalence varied between 1.40% and 19.09%. Furthermore, it showed that 13.00%-18.87% people examined in the Laboratory of Public Health in Macao were infected with *C. sinensis* between 1991 and 1997 [16]. It was estimated that 21 000 persons were infected with *C. sinensis* in Macao in 1995 [8]. Consequently, a conservative estimation of population with clonorchiasis in China was 12.93 million in 2004 (Table 1). It was similar to the estimation of 15 million by Lun *et al.* through analyzing the literature, but the data extracted from the literature spanned quite a long time and were discrepant from the nationally sampling survey in some areas [1].

**Table 1:** **The estimated population infected with*C. sinensis*globally in 2004**

**Region**		**1990s**^#^	**2004**			**Note**
			**Male**	**Female**	**Both**	
**China**	**Mainland**	4701000	7870000	4620000	12490000	Reference [10] and [11]. The infected of the male and female is deduced from the sex ration of 1.0674 [14] and the respective prevalence of 2.94% and 1.84% [10,11].
	**Taiwan**	NK	56155	32965	89120	The total infected is deduced from a conservative prevalence of 0.40% [1] and the total population of 22.28 million [14]. The estimation for different sexes refers to that in mainland of China.
	**Hong Kong**	333000	209825	123175	333000	The total infected adopts the estimation by WHO report in 1995 [8] and the estimation for different sexes refers to that in mainland of China.
	**Macao**	21000	13232	7768	21000	
**the Republic of Korea**		950000	782383	391841	1174224	Reference [17].
**Vietnam**		1000000	630104	369896	1000000	The total infected adopts the estimation by WHO report in 1995 [8] and the estimation for different sexes refers to that in mainland of China.
**Russia**		3000	1890	1110	3000	
**Total**		7008000	9563589	5546755	15110344	

^#^ According to the figure issued by WHO in 1995 [8]. NK: Not known.

In the Republic of Korea, clonorchiasis has been becoming a predominant parasitic disease recently. According to the nationally sampling survey in 2004, the total population infected with helminthiases was 1.78 million, of which 1.17 million was with clonorchiasis [17]. In addition, another large scale survey along the 4 major rivers in southern areas in 2006 showed the prevalence reached 17.1% in Nakdong-gang, 11.2% in Seomjin-gang, 5.5% in Youngsan-gang and 4.6% in Guem-gang [18].

Vietnam is another important endemic area for liver flukes, where clonorchiasis and opisthorchiasis coexist [19]. It was estimated that 1 million people in Vietnam were infected with clonorchiasis in 1995 [8]. According to the newest report by De *et al.*, clonorchiasis is endemic in 21 northern provinces, while opisthorchiasis in 11 southern provinces [20]. The prevalence of clonorchiasis in northern areas varied from 0.2% to 40.1%. Nevertheless, no nationally sampling survey has yet been implemented and most of the surveys were small scale and non-sampled [19]. Furthermore, it is argued many so-called *C. sinensis* infected cases are actually infected with other intestinal trematodes [21]. Thus, it is challenging to arrive at an exact figure of population infected with clonorchiasis in Vietnam. However, taking into consideration of such factors as a huge population at risk (33 million [22]), local habit of eating raw or undercooked fish [21,23], and rapid growth of freshwater fish production [2], 1 million people estimated to be infected with clonorchiasis should not be unreasonable.

*C. sinensis* is also endemic in far east area of Russia and the population infected estimated by WHO in 1995 were about 3 000 [8]. Additionally, 1.22 million people were estimated to be infected with *O. felineus*[8]. However, the current status in Russia remains unclear.

Recently, eggs of *C. sinensis* were detected from human feces through PCR-based method in *O. viverrini* endemic area of Thailand [24]. It was found only 64% individuals were infected with *O. viverrini* and additional 23% with *C. sinensis*. This extended the traditional knowledge of clonorchiasis. Therefore, whether *C. sinensis* is also endemic in other traditional endemic areas of *O. viverrini* or *O. felineus* deserves more attention and *vice versa*.

Little is known on the epidemiological situation of clonorchiasis in Democratic People’s Republic of Korea (DPRK). To our best knowledge, only one article has attempted to explore this problem [25]. Out of 137 patients from a hospital in Cheongjin-shi, Hamgyeongbuk-do, DPRK, 27 were positive for clonorchiasis using ELISA test. Furthermore, among 133 female immigrants from DPRK to the Republic of Korea, 4 were with clonorchiasis by the same test. Because clonorchiasis was highly endemic in the northeast of China and the Republic of Korea both neighboring to DPRK, and the Korean nationality in the former two countries loves eating raw fish [10,11,17,18], it is probable that clonorchiasis is also an important parasitic disease in DPRK. However, it is difficult to predict the epidemiological situation.

Dozens of clonorchiasis cases have been documented in Malaysia [26-28]. In addition to human cases, infections in cats and farmed fish were also reported [29-31]. However, two local snail species - most likely to act as the intermediate host - were not susceptible to infection [29]. Therefore, local transmission is remaining controversial. Nevertheless, such disease should not be neglected due to high popularity of eating raw fish in traditional festivals [27].

During 1947–1950, *C. sinensis* occurred in 19 prefectures of Japan. Owing to integrated control programme, clonorchiasis has been successfully controlled. In 1991, no case was found in 1 million stool samples examined [8]. Nowadays, only occasional cases were reported [32,33]. Apparently, clonorchiasis is no longer endemic in Japan.

There are also case reports in other parts of Asia [34,35], Europe [36-46], North America [47,48], South America [49-51], Australia [52] and even Africa [53]. Those reported cases usually were symptomatic, even with severe complications. Due to the lack of knowledge among population and medical organizations and reports bias-tendency to report those with complications, the actual number of persons infected should be higher. The reported cases were mainly immigrants from endemic countries or travelers who had visited endemic countries and eaten raw or undercooked fish.

In summary, clonorchiasis was predominantly endemic in China, the Republic of Korea, Vietnam and part of Russia. It is also probably endemic in DPRK and possibly in Malaysia. The situation in other traditional *O. viverrini* or *O. felineus* endemic areas is not yet clear. Sporadic human cases are also reported from other countries either due to international travelling or owing to immigration. The conservative estimation of population infected with *C. sinensis* reached 15 million in 2004 globally, of which over 85% were in China (Table 1).

However, the figure of clonorchiasis in the globe may be complicated by several reasons. First, the eggs of *C. sinensis*, *O. viverrini* and *O. felineus* are morphologically similar and difficult to be distinguished under microscopic examination [3,54,55]. Based on recently developed PCR test, *C. sinensis* was also detected in traditional *O. viverrini* endemic areas [24]. Therefore, *C. sinensis* is probably occurring in traditional endemic areas of *O. viverrini* and *O. felineus*, which was neglected previously and *vice versa*. In addition, some minute intestinal flukes whose eggs assemble that of *C. sinensis* also complicate the diagnosis of clonorchiasis [55]. Second, examination techniques contribute to the underestimation of prevalence of clonorchiasis. Current diagnoses are mainly based on fecal examination which is the “gold” standard, particularly Kato-Katz technique and formalin ether technique. However, many studies showed there existed false negative result using these techniques for schistosome, soil-transmitted helminths, as well as *O. viverrini*, especially in low infection intensity [56-61]. Third, early data pertaining to clonorchiasis in some areas may also cause underestimation. Due to the usual resistance to modifying food habit and the growing population in endemic areas, it is reasonable to assume that the population infected is increasing [7]. However, the early data in 1990s were employed to estimate the status of clonorchiasis in Hong Kong, Macao, Vietnam and Russia. Furthermore, the potential endemicity in DPRK and Malaysia is excluded from the estimation.

### Increasing trend in prevalence

Compared to the estimation of 7 million population infected with clonorchiasis in 1990s [8], the number in 2004 had doubled. Drastic increase occurred in mainland of China, from 4.70 million to 12.49 million [8,10,11]. The prevalence in the Republic of Korea fluctuated, which declined from 4.6% in 1971 to 1.4% in 1997 and bounced to 2.4% in 2004 [17]. The pattern in Vietnam and other areas is unclear. In the endemic areas, on the one hand it is difficult for the elder preferring eating raw fish to modify diet habit, and on the other hand the younger gradually get accustomed to this habit. Furthermore, the growth of population and freshwater fish aquaculture are other important contributors. There was a growth of 102 million population in the three major endemic areas, namely China, the Republic of Korea and Vietnam from 1995 to 2004 [62]. Meanwhile, the production of freshwater fish has increased by 55%, 116% and 91% in the aforementioned countries [63].

### Variability among sexes and age

**Figure 2:**
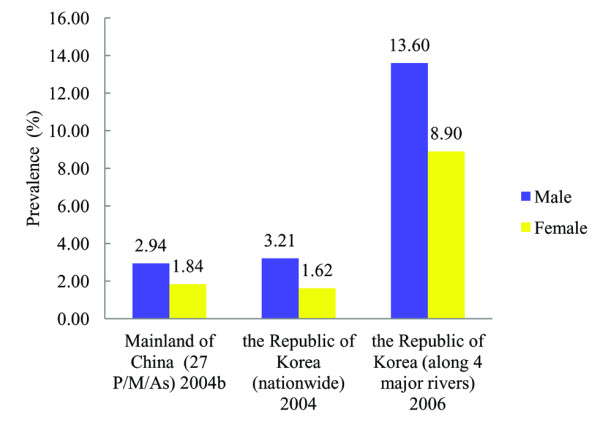
**The variance of prevalence of clonorchiasis by sexes in mainland of China and the Republic of Korea**.

The prevalence in the male is generally higher than that in the female. According to the special survey in 2004b in endemic areas of mainland of China, the prevalence was 2.94% in the male, while it was 1.84% in the female (Figure 2) [10,11]. The prevalence was 3.21% and 1.62% in the male and female respectively in the Republic of Korea’s national survey in 2004 [17], and 13.60% and 8.90% along 4 major rivers in 2006 [18]. Similarly, it was found that the prevalence in the male was 2–3 times or more than that in the female in local surveys in Vietnam [19]. Accordingly, among 15 million population infected, 9.6 million are male and 5.5 million are female (Table 1). The infection intensity is also higher in the male than that in the female. Taking the special survey in mainland of China in 2004b for example, those with moderate and heavy intensity (eggs per gram of feces ≥ 1 000) accounted for 28% in the male, while it was only 15% in the female [10,11].

**Figure 3:**
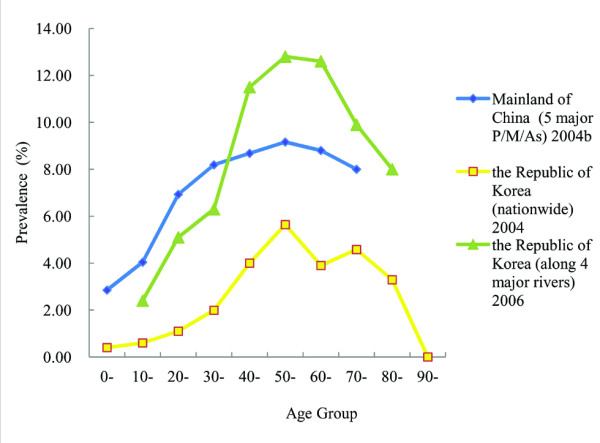
**The variance of prevalence of clonorchiasis by age in mainland of China and the Republic of Korea**.

The prevalence increases with age and reaches the highest in the age group of 50–59 (Figure 3) [10,11,17,18] or 40–49 (in local surveys in Vietnam) [19]. Similarly, the infection intensity also reaches the peak in the age group of 50–59 [10,11].

The distribution characteristics among sexes and age are mainly related to social customs, in other words, human raw-fish-eating behavior, and no yet evidence of concomitant immunity has been found to play important roles [64]. Raw fish is often consumed at restaurants or social parties, which the male have more chance to participate in [11,23,64,65]. Moreover, raw fish are often enjoyed over alcohol, which is more common among the male than the female and excludes children [7,64]. Furthermore, adult worms can survive in the body for decades [52,64]. Consequently, the exposure of the adult male is higher and so does the worm load, which leads to higher prevalence and infection intensity. However, in some local high endemic areas (prevalence over 60%), there is no significant difference in prevalence by sexes [66,67]. The decline of prevalence in those older than 60 (50 in local surveys of Vietnam) is probably due to the early death caused by clonorchiasis-related complications [68]. We also argue that elderlies seek for medical services more frequently due to clonorchiasis-related complications or unrelated diseases, and then accept diagnosis and treatment. It’s not surprising that the peak of *O. viverrini* infection also occurs in the age group of 50–59 in Thailand-the most endemic area of *O. viverrini* with an overall prevalence of 8.7% and over 6 million infected [22,69,70]. Although at low level, some children is also infected. In some areas of China, children like to ingest incompletely roasted small fish and get infected [10]. In the Republic of Korea, sometimes children are given raw fish by their mothers who think raw fish can make their children strong in traditional ideas [64,71]. However, in other areas, children are usually not allowed to eat raw fish [23].

### Endemicity

**Figure 4:**
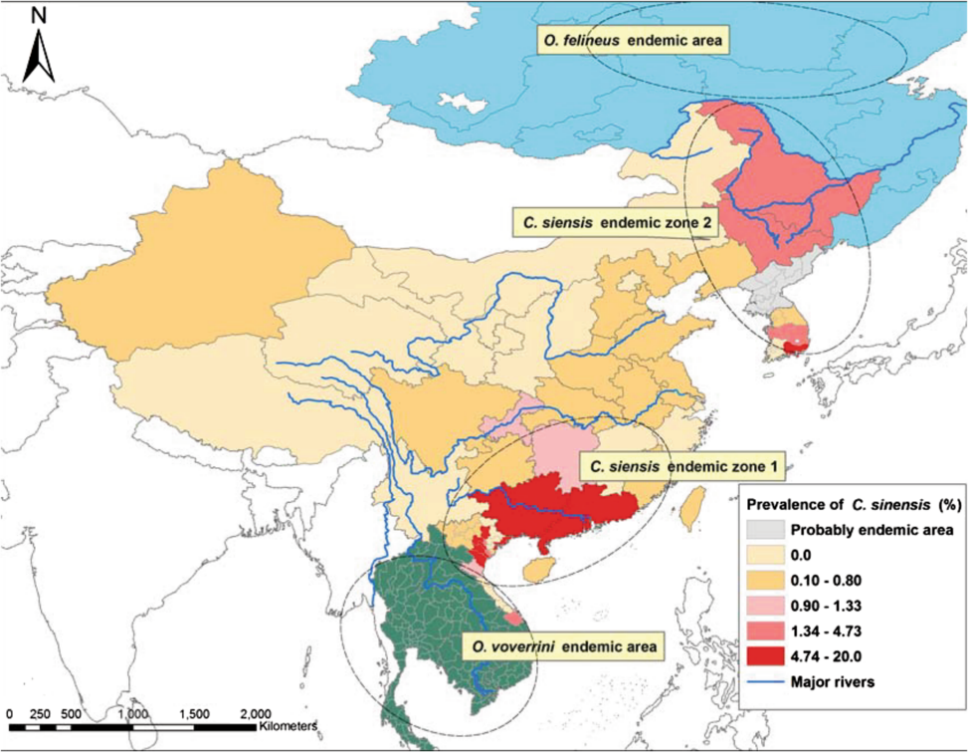
**The global distribution of three major species of liver flukes, i.e.*C. sinensis*, *O. viverrini *and *O. felineus*.** This map focuses on the endemicity of *C. sinensis* in China, the Republic of Korea and the northern part of Vietnam.

Due to different distribution of intermediate host and food habit, the distribution of clonorchiasis also varies in different areas, measured by endemicity. In a global view, it mainly distributes in China, the Republic of Korea and Vietnam. However, the prevalence is various even in the same country. There are two major endemic regions in China, i.e. the southeast including Guangdong and Guangxi, and the northeast including Heilongjiang, Jilin and Liaoning, with the prevalence of 16.42%, 9.76%, 4.73%, 2.90% and 0.80%, respectively (Figure 4) [10,11]. The top four endemic areas in the Republic of Korea are Gyeongsangnam-do (11.3%), Daejeon (6.9%), Chungcheongnam-do (6.8%) and Jeollanam-do (6.2%),while there is no infection in Jeju-do (Figure 4) [17]. To current knowledge, clonorchiasis is endemic in 21 northern provinces of Vietnam (Figure 4) [20].

Thus, the distribution range of *C. sinensis* is mainly consisted of two epidemic zones. The first zone includes the southeast of China and the northern area of Vietnam, and the second one covers the northeast of China, the Republic of Korea, part of Russia and probably DPRK (Figure 4). The first zone neighbors to the endemic area of *O. viverrini* (mainly in Thailand, Laos, Cambodia and the southern part of Vietnam [2,4,8,19,20,22,69,70]), while the second one is adjacent to that of *O. felineus* (mainly in Russia [2,4,8,72]).

## Association of *C. sinensis* infection with CCA

CCA, usually with poor prognosis, is a primary hepatic malignancy arising from bile duct epithelium and ranks the second most common primary hepatic malignancy [73-75]. The incidence of CCA varies greatly among different areas of the world and so does the constituent ratio of CCA in hepatic malignancy, which is related to the distribution of risk factors [76,77]. Liver fluke infection is just one important factor [74,76-78]. In 1994, *C. sinensis* and *O. viverrini* were classified as “probably carcinogenic to humans” (Group 2A) and “carcinogenic to humans” (Group 1) by IARC, respectively [79]. Based on more evidences, *C. sinensis* is reassessed as “carcinogenic to humans” (Group 1) in 2009 [5,6].

### The epidemiological studies

Many reports on *C. sinensis* infection-related CCA have been documented. Cited here are 8 cross-sectional or case–control studies and relevant important information is extracted and listed in Additional file 2[80-87].

During 1960s and 1970s, three cross-sectional studies were carried out in Hong Kong and the Republic of Korea, which preliminarily showed the probable carcinogenesis of *C. sinensis* infection to human (Group 2A) [80-82]. Three case–control studies in the Republic of Korea were then published between 1996 and 2008, which promoted the reassessment of it as “carcinogenic to humans” (Group 1) in 2009 [83-85]. Recently, another two case–control studies have also been reported in mainland of China, which further demonstrated the etiological relationship [86,87].

Some aspects deserve being emphasized. Firstly, ideally, the case and control represent the source population, otherwise there exists bias. *C. sinensis* infection mainly causes liver and biliary diseases, of which gallstone is very frequent [88,89]. Thus, the criteria for inclusion and exclusion of subjects are important. However, in one cross-sectional study, the control consisted of those with liver disease, which biased to include those with *C. sinensis* infection [81]. In another case–control study, persons with stones in the bile ducts were excluded from the case, which biased to exclude those infected with *C. sinensis*[84]. Furthermore, in the same case–control study, many patients with alcohol induced pancreatitis were included as controls although they didn’t have hepatobiliary diseases. However, it may also bias to include those with *C. sinensis* infection in the control. On the one hand, *C. sinensis* infection can induce pancreatitis [3,90-95]. On the other hand, raw-fish-eating habit is associated with alcohol drinking [7,64]. Therefore, the relationship intensity (odds ratio, OR) may be underestimated in above two studies. Secondly, the determination of exposure is another important problem, which may cause misclassification bias. When stool examination is adopted, the misclassification is prone to occur in the case, because bile obstruction usually occurs in CCA cases. For example, bile obstruction occurred in more than half of cases in one study, which led to contrary results when stool examination and pathological examination were adopted, respectively [84]. Additionally, the development of cancers is chronic with a long process and past exposure may also be risk factor. Therefore, serologic test and radiologic examination may be more sensitive, as past infection can also be detected [84]. It is regretting that no definite method for determining exposure was mentioned for the two studies in mainland of China [86,87].

In addition to the studies listed in Additional file 2, one ecological study carried out in the Republic of Korea also showed the positive relationship [96]. The prevalence of clonorchiasis in Chuncheon, Chungju and Haman was 2.1%, 7.8% and 31.3%, respectively, while the incidence of CCA was 0.3/100 000, 1.8/100 000 and 5.5/100 000, the proportion of CCA in liver cancer was 1.4%, 8.9% and 13.2%, respectively. Furthermore, another case–control study was reported in mainland of China, but *C. sinensis* infection had only been tested in cases rather than controls [97].

### Estimation of CCA cases attributable to *C. sinensis* infection

The estimation of CCA cases attributed to *C. sinensis* infection can be based on either formula below: [98,99]

(1) CCAs attributed toC.sinensis=population infected×CCA incidence of uninfected×(odds ratio−1)

(2) $$\text{CCAs attributedÂ to}\;C.\sin ensis=\text{totalÂ CCAsÂ }\times \left\{\frac{C.\sin ensis\;prevalence\;rate*\left(odds\;ratio-1\right)}{\left[C.\sin ensis\;prevalence\;rate*\left(odds\;ratio-1\right)\right]+1}*100\%\right]$$

Although different parameters are necessary in formula (1) and formula (2), the prevalence of *C. sinensis* and the population infected could be converted mutually. Obviously, no matter which formula is applied, the relationship intensity, namely OR, is of crucial importance.

**Figure 5:**
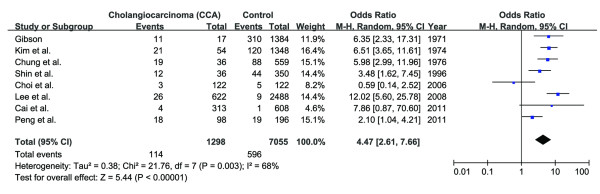
**Meta-analysis of relationship intensity (OR) between *C. sinensis *infection and CCA.** Events denote *C. sinensis* infection.

Based on the studies shown in Additional file 2, the comprehensive OR can be deduced through meta-analysis. Original OR rather than adjusted one in each study is applied, because those studies had different objectives and designs and took into consideration of different aspects. The overall OR is 4.47 (95% CI: 2.61-7.66) (RevMan software, http://ims.cochrane.org/revman) (Figure 5). Other researchers have done similar analysis. It was 4.65 (95% CI: 2.21-9.79) through meta-analysis of the related studies in the Republic of Korea [99], while it was 4.84 (95% CI: 2.79-8.41) after including those studies pertaining to *O. viverrini* infection and CCA [77,100-103].

Shin *et al.* have managed to estimate clonorchiasis-induced CCA in the Republic of Korea [99]. Due to lack of CCA data in other *C. sinensis* endemic areas, that in the Republic of Korea will be extrapolated (Table 2). Firstly, based on formula (2), the number of CCA cases in the Republic of Korea (2 166 male and 1 440 female in 2005 [99]), *C. sinensis* prevalence (3.7% in the male and 1.6% in the female in 1981 [17]) and the overall OR (4.47), it is estimated that 246 male and 76 female CCA cases attributed to *C. sinensis* infection occurred in the Republic of Korea in 2005. Secondly, according to the clonorchiasis-related CCA cases occurring in the Republic of Korea in 2005 and the population infected with *C. sinensis* in 1981, the incidence of CCA among *C. sinensis* infected population is deduced, namely 35/100 000 and 25/100 000 in the male and female, respectively. Thirdly, after multiplying the global population infected, the global CCA cases attributed to *C. sinensis* infection are captured, namely 4 726, of which 3 345 are male and 1 381 are female (Table 2). It must be cautious that the morbidity of cancer is a chronic progress spanning several decades. Thus, the prevalence of *C. sinensis* in 1981 was adopted, while CCA cases in 2005 were applied [99]. Likewise, the deduced global clonorchiasis-related CCA cases may occur several decades later theoretically.

**Table 2:** **The estimated incidence of CCA among population infected with*C. sinensis*and CCA cases attributable to*C. sinensis*infection globally**

**Sex**	**Total CCA cases in the Republic of Korea in 2005**^#^	*C. sinensis *prevalence in the Republic of Korea in 1981^*^	**OR (95% CI)**	**CCA cases attributable to *C. sinensis *infection in the Republic of Korea (95% CI)**	**Population in the Republic of Korea in 1981**^★^	**Population infected in the Republic of Korea in 1981**	**CCA incidence in infected (per 100 000) (95% CI)**	**Global population infected in 2004**	**CCA cases attributable to *C. sinensis *infection Globally (95% CI)**
**Male**	2166	3.70%	4.47 (2.61-7.66)	246 (122–428)	19041203	704525	35 (17–61)	9563589	3345 (1653–5813)
**Female**	1440	1.60%	4.47 (2.61-7.66)	76 (36–139)	19013400	304214	25 (12–46)	5546755	1381 (659–2528)
**Total**	-	-	-	-	-	-	-	-	4726 (2312–8341)

^#^Reference [99]; ^*^Reference [17]; ^★^Reference [62].

One obvious limitation is that no individual OR has been available for the male and female, respectively. In the study focusing on the relationship between *O. viverrini* infection and CCA, it was found the higher the infection intensity, the higher the OR [102]. Furthermore, compared to the female, the male infected have higher infection intensity as was depicted above [10,11]. Thus, it is reasonable to assume different ORs should be offered for different sexes. However, due to lack of such data, only the same OR is adopted here.

## Conclusion

A conservative estimation was made that 15 million people were infected with *C. sinensis* in 2004 in east Asia, especially in China, the Republic of Korea and Vietnam, which may cause nearly 5 000 CCA cases annually in the future. Although the population infected is relatively smaller compared to schistosomiasis and soil-transmitted helminthiases, the distribution is highly concentrated in several Asian countries, where the public health is being threatened severely. Furthermore, the impact is increasing and expanding to non-endemic areas due to the growth and movement of population and the rapid development of aquaculture. Thus, it is listed among the most neglected tropical diseases [7,104]. Fortunately, the evaluation on the burden of food-borne diseases was launched by WHO in 2006 and clonorchiasis and other liver fluke diseases were included [105]. The assessment of disease burden promotes the awareness of harm, adoption of intervention and evaluation of cost-effectiveness. Epidemiological data are the basis of assessment of disease burden. Only when the data are available, can disease burden be calculated objectively. CCA is the most severe and fatal outcome caused by *C. sinensis* infection, which predominantly constitutes the mortality lost of disease burden in term of disability-adjusted life years [106]. Therefore, the epidemiological data on CCA is also crucial. It is expected the deduced data on population infected and CCA cases here will benefit the evaluation of disease burden of clonorchiasis, which will promote the control and prevention ultimately.

However, further researches on epidemiology of clonorchiasis and CCA should be carried out. Although nation-wide surveys have been carried out in China and the Republic of Korea, the epidemiological status in Vietnam is not yet clear and thus nationally sampling survey would be expected. Whether there exist local transmission and even epidemicity in DPRK and Malaysia also needs to be solved. Another important issue is the species discrimination, which may turn to the molecular biology techniques. Now that Kato-Katz technique is still the major diagnosis method as it is pathogen-oriented, convenient and at low cost, how to reduce the underestimation especially in low infection level is important. The accurate evaluation of prevalence will promote the adoption of suitable intervention and objective evaluation of disease burden. New techniques such as model simulation may deserve being introduced. Further studies on the relationship between *C. sinensis* infection and CCA are also expected, especially the separate OR for different sexes. The determination of exposure should also be further approached. The progress of CCA is chronic and complicated in which various factors are involved. Consequently, the public health impact of clonorchiasis is remarkably indicated by the above mentioned result that the deduced CCA cases are significantly attributed to *C. sinensis* infection with an overall OR of 4.47, although the result here may not be highly accurate. As it should be, new methods are expected to be applied and more accurate data to be captured.

## Competing interests

The authors declare that they have no competing interests.

## Authors’ contributions

M-BQ and X-NZ designed the study. M-BQ and Y-DC collected the data. M-BQ, SL and G-JY analyzed the data. M-BQ and X-NZ wrote the paper. All authors read and approved the manuscript.

## Supplementary Material

Additional file 1 Multilingual abstracts in the six official working languages of the United Nations. Click here for file

Additional file 2 **Epidemiological studies on the relationship between *C. sinensis *infection and CCA.** Click here for file
