# Supplementary material for: The global epidemiology of clonorchiasis and its relation with cholangiocarcinoma
Source: Infect Dis Poverty. 2012 Oct 25;1:4. doi: 10.1186/2049-9957-1-4 (PMC3710150; doi:10.1186/2049-9957-1-4)
Supplement: Additional file 1 — Multilingual abstracts in the six official working languages of the United Nations. [file 2049-9957-1-4-S1.pdf]

Translation of the abstract into the six official working languages of the United Nations

علم الأوبئة العالمية وداء متفرعات الخصية وعلاقته بسرطان القنوات الصفراوية

مان-باو كيان، يينغ-دان تشن، سونغ ليانغ، جو-جينج يانغ، وشياو-نونغ تشو

ملخص

تتناول هذه الورقة البحثية الوضع الوبائي وخصائص داء متفرعات الخصية على المستوى العالمي والعلاقة المسببة بين عدوى متفرع الخصية الصيني وسرطان القنوات الصفراوية (CCA). ويقدر عدد المصابين في عام 2004 بحوالي 15 مليون شخص على مستوى العالم، أكثر من 85٪ من حالات العدوى تم رصدها في الصين. ويزداد انتشار وبائيات داء متفرعات الخصية بصورة متباينة بين الجنسين بغض النظر عن السن، ويتركز في مناطق متفرقة جغرافياً. وتشير بيانات أخرى إلى أن عدوى C الصينية يُعد عدوى مسرطنة للإنسان، ومن المتوقع أن يصل عدد حالات الـCCA الناجمة عن عدوى C الصينية إلى 5000 حالة سنوياً في العشيرين سنة القادمة، بالإضافة إلى نسبة 4.47 يُصبح داء متفرعات الخصية أحد أهم المشكلات الصحية العامة في شرق آسيا، وبالتالي فإنه من الجدير بالأهمية الاستمرار في إجراء الدراسات الوبائية.

Translated from English version into Arabic by Dina El Kassas, through

TRANSLATORS WITHOUT BORDERS

华支睾吸虫病全球流行情况及其与胆管癌的关系

钱门宝，陈颖丹，梁松，杨国静，周晓农

摘要

本文就全球华支睾吸虫病的流行情况和特征、华支睾吸虫感染与胆管癌的病因学关系进行综述。保守估计 2004 年全球有 1 500 万人感染华支睾吸虫，其中超过 85% 在中国。华支睾吸虫病流行特征表现为上升趋势、性别和年龄差异及地区差异。越来越多的证据表明华支睾吸虫感染是人类的致癌因素。与非感染者相比，华支睾吸虫感染者罹患胆管癌比值比为 4.47。估计几十年后，全球每年可能有近 5 000 例由华支睾吸虫感染所致的胆管癌病例。华支睾吸虫病正在成为东亚的一个重要公共卫生问题，需要对其开展更深入的流行病学研究。

Translated from English version into Chinese by Qian Men-Bao

L’Épidémiologie Mondiale de la Clonorchiase et Son Lien avec le Cholangiocarcinome

Men-Bao Qian, Ying-Dan Chen, Song Liang, Guo-Jing Yang et Xiao-Nong Zhou

Résumé

Ce document examine le statut et les caractéristiques épidémiologiques de la clonorchiase à l’échelle mondiale et le lien étiologique entre l’infection par le *Clonorchis sinensis* et le cholangiocarcinome (CCK). Selon une estimation prudente, 15 millions de personnes dans le monde ont été infectées en 2004, dont plus de 85% en Chine. L’épidémiologie de la clonorchiase se caractérise par la tendance croissante de sa prévalence, sa variabilité selon l’âge et le sexe, ainsi que par son endémicité dans différentes régions. Des données supplémentaires indiquent que l’infection par le *C. sinensis* est cancérogène chez les êtres humains, et l’on prévoit que près de 5 000 cas de CCK attribués à l’infection du *C. sinensis* pourraient survenir annuellement dans le monde entier après plusieurs décennies, avec son rapport des cotes global de 4,47. La Clonorchiase est en passe de devenir l’un des problèmes de santé publique majeurs en Asie de l’Est et il serait utile d’entreprendre des études épidémiologiques plus approfondies.

Translated from English version into French, through

TRANSLATORS
WITHOUT BORDERS

Всемирная эпидемия клонорхоза и как это связано с холангиокарценомой

Мэн-Бао Цян, Ин-Дан Чен, Сун Лян, Го-Цзин Ян и Сяо-Нун Чжоу

Резюме

Эта работа рассматривает эпидемиологическое состояние и характеристику клонорхоза на мировом уровне, а также – этиологическую связь между инфекцией клонорхоза (*Clonorchis sinensis*) и холангиокарценомой (CCA). По оценкам исследования (на 2004 год) выяснилось, что минимум 15 миллионов людей во всём мире уже инфецировано, а больше 85% из их числа проживают в Китае. Тенденция роста эпидемии клонорхоза характеризуется распространением между мужчинами и женщинами любого возраста, причём в различных регионах. Многие данные показывают, что инфекция клонорхоза (*C. sinensis)* карценогенна по отношению к человеку. Отсюда вытекает предположение, что несколькимим десятилетиями позже, ежегодно во всём мире будет выявляться около пяти тысяч случаев CCA, относящихся к инфекции *C. Sinensis,* где ОШ составит в общем 4.47. Клонорхоз становиться одной из самых больших проблем здравоохранения в Восточной Азии, поэтому стоит и в дальнейшем проводить эпидемиологические исследования в этой области.

Translated from English version into Russian by Olga van der Veen, through

TRANSLATORS
WITHOUT BORDERS

La Epidemiología Global de la Clonorquiasis y su relación con el Colangiocarcinoma

Men-Bao Qian, Ying-Dan Chen, Song Liang, Guo-Jing Yang and Xiao-Nong Zhou

Resumen

Este artículo examina el estado epidemiológico y las características de la clonorquiasis a nivel global, así como la relación etiológica entre la infección *Clonorchis sinensis* y el colangiocarcinoma (CCA). Según una estimación conservadora se calculó que 15 millones de personas estaban infectadas en el mundo en 2004, de los cuales el 85% se encontraba en China. La epidemiología de la clonorquiasis se caracteriza por una tendencia al alza en su prevalencia, variabilidad entre sexos y edades, así como la endemicidad en diferentes regiones. Otros datos indican que la infección *C. sinensis* es carcinógena y se predice que se podrían dar cerca de 5000 casos de CCA anuales en el mundo atribuidos a la infección *C sinensis* décadas más tarde, con un índice de probabilidad total del 4,47. La clonorquiasis se está transformando en uno de los mayores problemas de salud pública en Asia Oriental y merece la pena llevar a cabo más estudios epidemiológicos.

Translated from English version into Spanish by Daniel Gonzalez, through

TRANSLATORS
WITHOUT BORDERS
